# ALSA: Associative Learning Based Supervised Learning Algorithm for SNN

**DOI:** 10.3389/fnins.2022.838832

**Published:** 2022-03-31

**Authors:** Lingfei Mo, Gang Wang, Erhong Long, Mingsong Zhuo

**Affiliations:** FutureX LAB, School of Instrument Science and Engineering, Southeast University, Nanjing, China

**Keywords:** spiking neural network, associative learning, supervised learning, STDP, long-term plasticity

## Abstract

Spiking neural network (SNN) is considered to be the brain-like model that best conforms to the biological mechanism of the brain. Due to the non-differentiability of the spike, the training method of SNNs is still incomplete. This paper proposes a supervised learning method for SNNs based on associative learning: ALSA. The method is based on the associative learning mechanism, and its realization is similar to the animal conditioned reflex process, with strong physiological plausibility and rationality. This method uses improved spike-timing-dependent plasticity (STDP) rules, combined with a teacher layer to induct spikes of neurons, to strengthen synaptic connections between input spike patterns and specified output neurons, and weaken synaptic connections between unrelated patterns and unrelated output neurons. Based on ALSA, this paper also completed the supervised learning classification tasks of the IRIS dataset and the MNIST dataset, and achieved 95.7 and 91.58% recognition accuracy, respectively, which fully proves that ALSA is a feasible SNNs supervised learning method. The innovation of this paper is to establish a biological plausible supervised learning method for SNNs, which is based on the STDP learning rules and the associative learning mechanism that exists widely in animal training.

## Introduction

In recent years, neural networks have made great progress in the field of information processing. Especially with the development of deep neural network (DNNs) (Rawat and Wang, 2017) and convolutional neural networks (CNNs) (LeCun et al., 1989; O’Shea and Nash, 2015; Rawat and Wang, 2017), the performance and application range of artificial neural networks (ANNs) has been greatly improved. However, there are still some problems for the ANNs. For example, most ANNs train the network according to the backpropagation of errors. Therefore, ANNs training requires a large number of labeled samples which is labor-intensive. In addition, although ANNs claim to be physiologically plausible, their training process is different from biological neural networks, which are mainly based on gradient descent and error backpropagation. Different from biological neural networks, which are mainly based on unsupervised learning like Hebbian learning (Caporale and Dan, 2008), the learning process of ANNs is mainly based on supervised learning. At the same time, the error backpropagation mechanism commonly used in ANNs lacks widespread evidence in biological neural networks.

Spiking neural networks (SNNs) (Maass, 1997) attracts more and more researchers because of their similarity to biological neural networks (Pan et al., 2020; Zirkle and Rubchinsky, 2020). Compared with ANNs, SNNs uses spike rate or spike timing to transmit information between neurons (Maass, 1997) instead of using numerical values to transmit information, and its unsupervised training process is also based on physiologically plausible spike-timing-dependent plasticity (STDP) (Caporale and Dan, 2008; Diehl and Cook, 2015; Masquelier and Kheradpisheh, 2018; Mozafari et al., 2019) instead of error backpropagation. Therefore, SNNs are closer to the biological neural network in principle. Thanks to the characteristics of SNN’s event-driven computing, those neurons that are not activated will not participate in the actual computing, thus saving computing resources. It is very suitable for low-power consumption computing on dedicated chips, such as TrueNorth (Akopyan et al., 2015), Tianjic (Pei et al., 2019), Loihi (Davies et al., 2018), Darwin (Shen et al., 2016), etc. Using these chips, SNNs have an order of magnitude advantage over ANNs in terms of computational power consumption (Akopyan et al., 2015; Davies et al., 2018; Pei et al., 2019; Xu et al., 2020).

The main reason restricting the development of SNNs is the lack of training algorithms, especially the supervised learning algorithms of SNNs. Since the spike signal is not differentiable, the error backpropagation widely used in ANNs cannot be used to train SNNs. At the same time, backpropagation also rarely exists in biological neural networks (Bengio, 2015; Lillicrap et al., 2020). Therefore, it is difficult to find a physiologically plausible SNN supervised training algorithm.

Many scholars have also proposed some training methods for SNNs, which can be mainly divided into the following two categories. The first is the ANN-SNN conversion. This type of method uses specific rules to convert the ANN trained networks into a corresponding structure of the SNN networks, making full use of the low power consumption characteristics of the SNNs calculation (Pérez-Carrasco et al., 2013; Diehl et al., 2015; Rueckauer et al., 2017). Since the training process does not occur in the SNNs, it cannot fully reflect the characteristics of the strong physiological rationality of the SNNs. The second type is based on error backpropagation to obtain higher model accuracy, such as Tempotron (Gütig and Sompolinsky, 2006), PSD(Yu et al., 2013), ReSuMe (Ponulak, 2005), MST (Gütig, 2016), EMLC(Yu et al., 2020), MPD-AL(Zhang et al., 2019), SpikeProp (Bohte et al., 2000), STCA(Gu et al., 2019), etc. The supervised learning methods mainly calculate the difference between the voltage of the output neuron at target time points and the threshold value to change the weight (Xie et al., 2016). There is also some research using backpropagation and gradient descent to train deep neural networks for SNN models (Lee et al., 2020). Most of these rules make proper adjustments to neurons or spike signals to make BP feasible in SNNs, but they lack certain physiological plausibility.

Compared with supervised learning, SNNs unsupervised learning is much more unified. At present, the most widely used unsupervised learning method of SNNs is STDP and its variants, which can obtain significant unsupervised clustering and feature extraction results (Diehl and Cook, 2015; Masquelier and Kheradpisheh, 2018; Tavanaei et al., 2019; Xu et al., 2020). At the same time, the STDP rules have been supported by many related experiments in the field of neuroscience. They have been widely confirmed in biological neural networks and have strong physiological plausibility (Caporale and Dan, 2008).

Almost all supervised learning rules use error backpropagation and gradient descent methods to achieve good accuracy, though these methods lack biological interpretability. Lee et al. (2015) pointed out that biological neurons are linear and non-linear operations, while backpropagation is purely linear, and there is no corresponding mechanism to realize the precise timing of backpropagation signals and the alternating of feedforward and feedback propagation, as well as retrograde signal propagation along axons and synapses. Therefore, there is no biological justification for back transmission. Lillicrap et al. (2020) also showed that while feedback connections are ubiquitous in the cortex, it’s hard to know how they transmit the error signals needed for backpropagation. The effect of feedback connection on neural activity still needs to be further explored. Given that, some researchers have begun to implement supervised learning combined with STDP rules. Legenstein et al. (2005) introduced a teacher signal by injecting current into the output neurons during training and combined it with STDP rules to achieve supervised learning. However, using this method does not guarantee STDP convergence for any input mode. The remote supervised method (ReSuMe) (Ponulak and Kasiński, 2010) uses STDP rules and makes output neurons spike at desired time points through a remote teacher signal. Wade (Wade et al., 2010) used Bienenstock, Cooper and Monroe (BCM) rules to adjust the learning window of STDP and proposed SWAT rules. In this method, the BCM model was used to slide the threshold and promote the synaptic weight to converge to a stable state. However, this method is only applicable to frequency coding. For ReSuMe and SWAT, although a liquid state machine or multi-layer feedforward network structure is used, only the synaptic weights of the output layer are learned, and the synapses of the hidden layer in the network are fixed after initialization. Hao et al. (2020) used symmetric STDP, combined with synaptic scaling and dynamic threshold, to achieve good results at both NMIST and fashion MNIST, but increasing the depth of the network has little effect on the performance of the network and is not conducive to the extension of this rule unless other methods such as convolution are introduced. Pfister et al. (2003), realized supervised learning by optimizing the possibility of observing postsynaptic impulse sequences at the expected time by starting from the criterion of probabilistic optimality and adding teacher signals to the model. But this model uses the relatively simple spike response model (SRM; Gerstner, 1995). Using other models will make this rule much more complicated. More details of probabilistic SNN can be found in Jang et al. (2019), which reviews probabilistic models and training methods based on a probabilistic signal processing framework. Also, there is some research that combines supervised and unsupervised STDP training. Using a simplified approximation of a conventional Bayesian neuron and an improved STPD rule, (Shrestha et al., 2017) combined unsupervised and supervised STDP learning to train a three-layer SNN on the MNIST dataset. Hao et al. (2020) achieved good performance on the MNIST dataset by combining their proposed symmetric spike-timing-dependent plasticity (sym-STDP) with synaptic scaling and dynamic threshold (Hao et al., 2020). There are also other plasticity-based unsupervised training with supervised modules (Querlioz et al., 2013; Kheradpisheh et al., 2018). However, most of these STDP based supervised learning methods do not have enough physiological plausibility.

To solve the above problems, an SNN supervised learning algorithm based on associative learning is proposed. The learning rules are based on the widely recognized STDP rules, and the classic STDP is simply adjusted while retaining physiological rationality. The major innovation of this paper is to establish a more biologically interpretable supervised learning method, which is based on the conditioned reflex associative learning mechanism that exists widely in animal training. To realize associative learning, an improved STDP model inspired by the heterosynaptic long-term plasticity is proposed.

The following contents of this article are mainly divided into methods, experiments and results, and discussion. The method part will introduce the neuron model, synaptic plasticity model, and the implementation methods of supervised learning. The experiments and results part includes the details of IRIS and MNIST classification networks, specific results, and process analysis of the classification tasks. In the discussion part, the advantages and current shortcomings of the supervised learning rules are analyzed.

## Methodology

### Neuron Model

In this paper, the leaky integrated and fire (LIF; Koch and Segev, 1998) model is adopted, which is a neuron model widely used in the field of SNN calculation and computational neuroscience simulation. This model is obtained by simplifying the Hodgkin Huxley (HH) (Hodgkin and Huxley, 1952) model but retains basic functionality. So that the computational results are close to those of the HH model, and the complexity and computational complexity of the model are greatly reduced. The model formula is shown as Equation 1.


(1) $$C_{m}\,\frac{d\,V}{d\,t}=-g_{L}\,\left(V-V_{L}\right)+I_{s\,y\,n}$$

Where *C_m_* is the membrane capacitance, *V*is the membrane potential, *g_L_* is the leakage conductance, *V_L_* is the leakage potential, and *I*_*syn*_ is the input current from the presynaptic neurons. Assuming that the total conductance value is *g_E_*, and the constant $\tau_{m}=\frac{C_{m}}{g_{L}}$ is defined, then (1) can be converted to (2).


(2) $$\tau_{m}\,\frac{d\,V}{d\,t}=-\left(V-V_{L}\right)-\frac{g_{E}}{g_{L}}\,\left(V-V_{E}\right)$$

(3) $$\tau_{E}\,\frac{d\,g_{E}}{d\,t}=-g_{E}+\sum_{j\in N_{E}}w_{j,i}\,\delta_{t}$$

*g_E_* in (2) will dynamically change under the influence of the presynaptic spikes, and the specific changes are shown in (3). That is, once the presynaptic neuron generates a spike, *g_E_* will increase non-linearly.*V_E_* is the reversal potential of excitatory neurons, τ_*E*_ is the conductance decay time constant of excitatory neurons, *N_E_* is the number of presynaptic neurons, δ_*t*_ is the specific moment when the presynaptic neuron generates spikes, and *w*_*j,i*_ represents the connection weight of presynaptic neuron j to postsynaptic neuron i.


(4) $$\text{if}\left(V>V_{t\,h\,r}\right)\left\{ \begin{array}{c} V=V_{L}\\ T_{r\,e\,f}=T_{0}\\ V_{t\,h\,r}=V_{t\,h\,r}+V_{t\,h\,r\,D\,e\,l\,t\,a} \end{array}\right.$$

**FIGURE 1:**
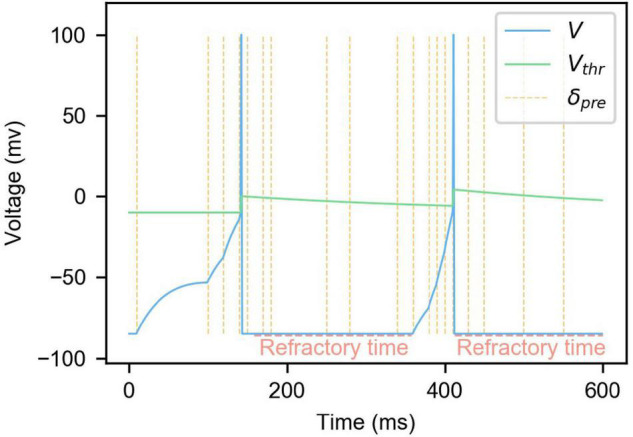
The changes of membrane potential and membrane potential threshold of LIF neuron model with dynamic threshold under the influence of presynaptic neuron spikes. In the figure, the blue curve is the neuron membrane potential, the green curve is the membrane potential threshold, and the yellow vertical dashed lines are the moments when the presynaptic spikes are fired.


As shown in (4), when the membrane potential V increases to exceed the membrane potential threshold *V*_*thr*_, the membrane potential will be reset, and the refractory period *T*_*ref*_ will be set to *T_0_*. During the refractory period, the neuron will not respond to the presynaptic spikes as shown in Figure 1. At the same time, in order to ensure that the spike firing frequency of neurons is stable in a specific range, and avoid the situation where some neurons are firing too much and others not enough, the mechanism of neuron dynamic threshold is introduced referring to Diehl’s approach (Diehl and Cook, 2015). Homeostasis, which is known in neuroscience, is also considered here (Watt and Desai, 2010). As shown in (4), every time a neuron generates a spike, the neuron threshold will be increased accordingly, thus raising the threshold for the next spike. *V*_*thrDelta*_ is a hyperparameter used to control the difficulty of neuron spike generation.

(5) $$\tau_{t\,h\,r}\,\frac{d\,V_{t\,h\,r}}{d\,t}=-\left(V_{t\,h\,r}-V_{t\,h\,r\,B\,a\,s\,e}\right)$$

Also, as shown in (5), *V*_*thr*_ will gradually decay to *V*_*thrBase*_, lowering the membrane potential threshold of neurons that do not produce spikes for a while. Combined with (4), the difficulty of neuron spike firing is controlled at a reasonable range. τ_*thr*_ is the dynamic threshold decay time constant. Figure 1 is an example of changes in neuron membrane potential and dynamic threshold. It can be seen from the figure that presynaptic neuron spikes will cause the neuron membrane potential to rise, and the membrane potential will slowly decrease over time. When the membrane potential exceeds the threshold, the membrane potential will rise and drop rapidly in a short time, completing the firing of a spike. Every time the neuron emits a spike, the membrane potential threshold will increase and gradually decay to its initial state. At the same time, when a neuron fires a spike, it will enter refractory time, during which the neuron does not respond to presynaptic spikes.

### Synapse Model

The synapse model used in this paper is mainly based on the STDP rule, and the classic STDP is appropriately adjusted to make it more in line with the needs of this model.


(6) $$△\,w=\left\{ \begin{array}{cc} \eta \left(\alpha +\beta \cdot e^{-\frac{I\,S\,I}{\tau_{p}}}\right)*w*\left(1-w\right)if\left(ISI>0\right) & \\ 0\;e\,l\,s\,e & \end{array}\right.$$

(7) $$I\,S\,I=t_{p\,o\,s\,t}-t_{p\,r\,e}$$

Equation 6 is the synaptic plasticity model used in this paper, where △*w* is the modified amplitude of the synapse weight after each spike, and ISI (inter-spike interval) is the time difference between the most recent spike time of the neuron before and after the synapse as in (7). η is the learning rate, α is a constant bias term that is usually less than 0 to simulate the heterosynaptic LTD (long-term depression)(Krug et al., 1985; Christie et al., 1994). β is used to adjust the intensity of weight change, usually greater than 0. τ_*p*_ is the time constant of the LTP (long-term potentiation) part to control the detail. Also, w(1-w) in equation 6 means limiting the weight to between 0 and 1. And when the current weight approaches 0 or 1, the change of weight is very small.

Heterosynaptic LTD is a long-term plasticity phenomenon that exists widely in biological neural networks (Krug et al., 1985; Christie et al., 1994). The main manifestation is that when a certain neuron generates a spike, the strength of the synapses which regards the current neuron as the postsynaptic neuron will be attenuated to a certain extent. This is mainly because VDCCs (voltage-dependent calcium channels) are activated after the neuron generates a spike signal. After the postsynaptic neuron pulses, the VDCC channel on the postsynaptic neuron opens, which activates inhibitory calmodulin such as PP1 in the cell, and produces a series of intermediate actions that ultimately lead to a decline in synaptic strength (Krug et al., 1985; Caporale and Dan, 2008). To realize heterosynaptic LTD, the classic STDP is modified in this paper. In the case of ISI > 0, LTP will be generated when ISI is less than a certain value, and LTD will be generated when ISI is greater than a certain value. In Equation 6, the parameter α simulates the heterosynapses, producing the results that in the case of ISI > 0, LTD is generated when ISI is greater than a certain threshold. When ISI < 0, if the same LTD as the classic STDP is used, then, on the whole, the effect of LTD will be much greater than that of LTP, which will make all synaptic weights tend to 0 in the process of training. Therefore, the delta weight was set to 0 when ISI < 0 to balance LTD and LTP. With this improved STDP and the heterosynaptic LTD, associative learning could be achieved.

### Supervised Learning Algorithm

**FIGURE 2:**
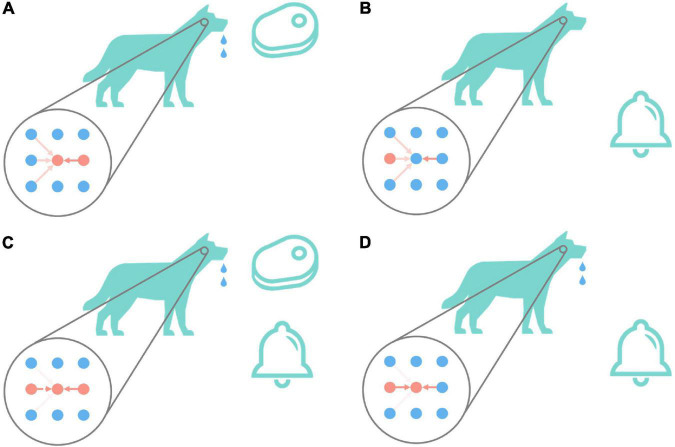
Pavlov’s dog experiment. The big circle in the figure represents the state diagram (imaginary) of the relevant neurons in the dog in the corresponding state. The first column represents auditory-related stimuli, the second column represents animal behaviors including drooling, and the third column represents olfactory-related stimuli. The depth of the red arrow indicates the strength of the excitatory connection between the corresponding neurons. The darker the color, the higher the strength of the connection between neurons, and vice versa, the lower the strength of the connection between neurons. The red dot indicates that the neuron is currently active (that is, it has fired a spike within a period), and the blue indicates that the neuron is resting (that is, it has not fired a spike for a while). The bells and meat in the picture will cause the second neuron in the first column and the second neuron in the third column to enter the active state, respectively. When the second neuron in the second column is active, the dog will drool. Pavlov’s dog experiment is conducted in the order of **(A–D)**.

Associative learning is the basis of cognition and plays an important role in the process of animal learning and training (McSweeney and Murphy, 2014). Figure 2 is the classic associative learning experiment of Pavlov’s dog (Pavlov, 2010). As shown in Figure 2A, in the beginning, the dog secretes saliva under the stimulation of meat. This is an instinctive behavior, that is, there are naturally high-strength connections between the meat neuron and drooling neuron. And as shown in Figure 2B, the dog does not drool under the stimulation of the bell, and the connections between auditory-related neurons and the drooling neuron are relatively weak, which cannot cause the dog to drool. As shown in Figure 2C, give dog meat and bell stimulation at the same time, repeat this step for a while, the connection between the bell and drooling neuron is gradually potentiated. The connections between other auditory neurons and drooling are weakened. The result is shown in Figure 2D. Only under the stimulation of the bell, the dog drools too. This process realizes the associated learning of bells and drooling.

The conclusion can be made by observing the changes in the connections between neurons in the process: The essence of associative learning is that the connection strength between neurons that are simultaneously activated within a period increases, and the connection strength between unrelated stimuli and unresponsive behaviors decreases. This phenomenon is also consistent with the Hebb rule “neurons that fire together, wire together.” The above steps are widely used in animal training to adjust the behavior of the training object by establishing the relationship between specific things (Pavlov, 2010). This process is similar to the effect achieved by supervised learning. So, is there a rule of synaptic long-term plasticity that can achieve similar effects, and then achieve associative learning and supervised learning?

**FIGURE 3:**
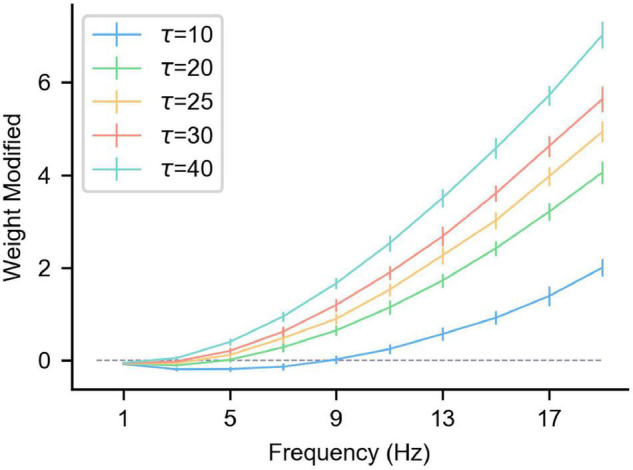
The influence of the pre/postsynaptic spike frequency on the synapse weight under different τ_*p*_. The gray dashed line in the figure is where the weight change is 0, and the length of the vertical lines on the curve represents the standard deviation of multiple trials (*n* = 50). The abscissa is the pre/postsynaptic spike frequency. Presynaptic and postsynaptic frequencies are equal and obey the Poisson distribution. Each simulation time is 100 s, α = –0.1, β = 1, and η = 0.01.

Figure 3 shows the △*w* of the synaptic long-term plasticity rule introduced above under different frequency pre/postsynaptic spikes. As can be seen from the figure, τ_*p*_ affects the results significantly. However, when the pre/postsynaptic spike frequency is high enough, △*w* under any τ_*p*_ tends to increase. To be specific, in multiple (*n* = 50) simulations, the pre-synaptic spikes obeyed the Poisson distribution at a specific frequency. The relative positions of the pre/postsynaptic spikes are uncertain, but the standard deviation of △*w* or multiple trials is controlled within a relatively small range (Figure 3), indicating that when the pre/postsynaptic spike frequency is high enough, the synaptic weight shows a relatively stable LTP. This result is consistent with experimental results in neuroscience (Sjöström et al., 2001). Therefore, when a specific spike pattern (a combination of neurons’ spike states, that is, some neurons generate spikes and others do not) is expressed in presynaptic neurons, synapses from presynaptic neurons relating a specific pattern to a specific postsynaptic neuron can be enhanced by inducing the specific postsynaptic neuron to fire. In the same way, the neurons that are not related to the current spike pattern do not produce spikes, and the strength of their connections to the current postsynaptic neurons is weakened under the effect of the heterosynaptic LTD. These characteristics can be used to enhance some synapses and weaken others, achieve a result similar to the above associative learning, and then realize supervised learning.

**FIGURE 4:**
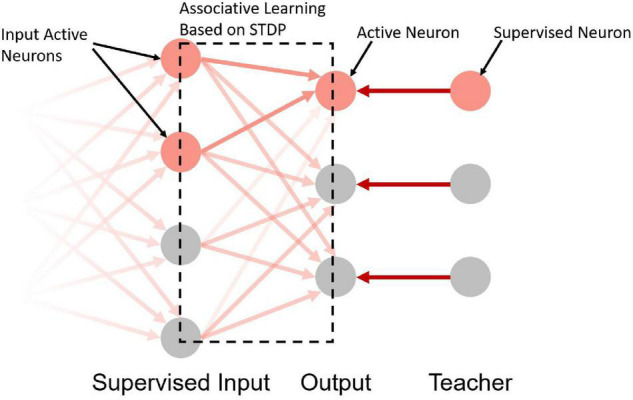
A schematic diagram of the supervised learning network structure. The first, second, and third layers are the supervised input layer, output layer, and teacher layer, respectively. The gradient on the left indicates that the first layer can be used as the output layer of the previous network.

Take the network in Figure 4 as an example. The input neurons in the network are equivalent to the bell signals in Figure 2, the output neurons in the network are equivalent to the drooling signals and the supervised neurons are equivalent to the meat signals. It simulates the associative learning process of Pavlov’s dog experiment, which enables the output neuron to learn the input signal based on both the teacher signal and the input signal.

The implementation steps of supervised learning are as follows:

(1) Construct the network structure shown in Figure 4, where the first column is the supervised learning input layer (can be used as the output layer of unsupervised learning or other supervised learning output layers). The second column is the output layer, and the third column is the teacher layer. The number of neurons in the output layer and the teacher layer is equal to the number of sample categories, and a one-to-one mapping relationship between the output layer, the teacher layer, and the sample categories is constructed.
(2) Input spike signals to the supervised input layer, the spike signals are from the encoded spikes or the spikes of the previous neurons. Mark all neurons with spikes in the supervised input layer as *I_s_* and others in the same layer as *I*_*non*_.
(3) Simultaneously with (2), input spike signals to the teacher layer of the corresponding category, and induce the output neurons of the corresponding category to generate spikes. Mark the neuron with spikes in the output layer as *O_s_* and others in the same layer as *O*_*non*_.
(4) Since *I_s_* and *O_s_* both emit spikes for a while, under the mechanism described above, as long as the spike frequency of *I_s_* and *O_s_* is high enough, the strength of the synaptic connection from *I_s_* to *O_s_* will increase. The specific enhancement intensities are positively correlated with the spike frequencies of each neuron in *I_s_*. At the same time, since *I*_*non*_ does not produce spikes, the strength of the synaptic connection from *I*_*non*_ to *O_s_* will decrease under the effect of the heterosynaptic LTD. The strengths of all synaptic connections to *O*_*non*_ remain unchanged.
(5) Change the next sample and label, repeat steps (2) to (4) until the training of all samples is completed.

In the inducement of association supervised learning, it is necessary to use the long-term plasticity rules introduced in Equations 6, 7. In contrast, due to the existence of the negative semi-axis LTD in the classic STDP, under the effect of high-frequency pre/postsynaptic spikes, the change of synaptic weights will have a greater relationship with the specific spike moments, which makes it difficult for stable LTP to arise as in Figure 3. At the same time, due to the lack of heterosynaptic LTD, the synaptic connection of unrelated neurons cannot be effectively inhibited, so it cannot be used to realize associative learning and supervised learning.

Based on the above phenomenon, the potentiation of specific neuron connections can be achieved by inducing target neurons to emit spikes, that is, the spike induction of target neurons can achieve synaptic potentiation between specific spike patterns and target neurons and synaptic depression between unrelated neurons and target neurons. We call it ALSA (associative learning based supervised learning algorithm for SNNs). The supervised part only exists in the teacher layer, which is realized by stimulating the neurons in the teacher layer with a certain frequency, and no additional statistics on the number of output spikes and the precise time of output spikes are required. Synaptic strengthening and weakening are still achieved through unsupervised long-term plasticity rules. Therefore, ALSA can be said to be more in line with biological interpretability which is based on the universal associative learning behavior of animals, and it is relatively easy to realize which requires only certain teacher stimulation. In the following part, the feasibility of ALSA will be verified by two specific experiments.

## Experiments and Results

The IRIS and the MNIST classification experiments are used as examples. The details and results of the experiments as well as the feasibility of ALSA are introduced in detail. The simulator we used is an event-driven high accurate simulator (EDHA) for SNNs (Mo et al., 2021).

### IRIS Classification

The IRIS (Dua and Graff, 2017) dataset contains three classes of irises, 50 of each class, and a total of 150 data. One of them is linearly separable from the other two, and the latter two are nonlinearly separable. The dataset contains four attributes: calyx length, calyx width, petal length, and petal width.

**FIGURE 5:**
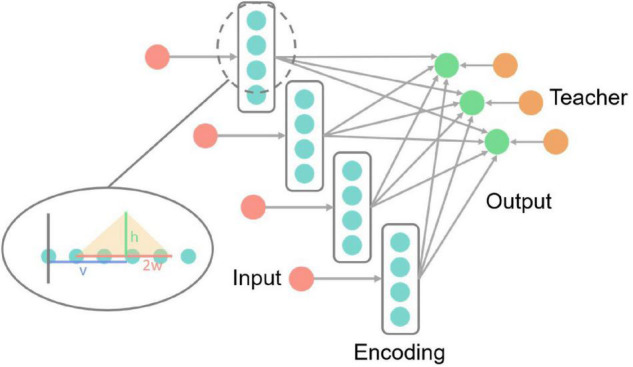
IRIS classification network structure diagram. Each red input neuron in the picture receives an input vector of the IRIS dataset and encodes the numerical information into the neuron spikes signal of the encoding layer. The three neurons in the output layer correspond to the three categories of samples in the IRIS dataset, and the teacher layer is to generate supervised signals. The enlarged part of the dotted line in the figure is the details of the neurons in the coding layer, and the yellow translucent triangle is the encoding triangle.


Figure 5 is the IRIS classification network structure diagram, including the input layer, encoding layer, output layer, and teacher layer. The input layer receives IRIS data and encodes it into neuron spikes of the encoding layer.

(8) $$f_{i}=\text{max}\,\left(0,\frac{-h\,\left|a_{i}-v\right|}{w}+h\right)$$

Since the input data of this dataset is all numerical information, it is difficult to directly use it in SNNs. Therefore, encoding is necessary. The encoding is realized by dispersing the data to multiple neurons. The encoding method is shown in (8), *f_i_* is the spike frequency of the corresponding subscript coding neuron, *a_i_* is the distance from the corresponding subscript neuron to the starting point (the gray vertical line in Figure 5), and the remaining variables are as circled in Figure 5 shown. v is the input value, w is one-half of the length of the bottom side of the encoding triangle, and h is the highest encoding spike frequency, that is, the height of the bottom side of the encoding triangle in Figure 5. Both w and h are hyperparameters. With the input value as the center, the closer the neuron is to the center of the input value, the higher the neuron spike frequency is.

Connections from the encoding layer to the output layer are fully-connected and all synapses are trainable. ALSA is implemented for training between the input layer and the output layer. The three neurons in the output layer correspond to three categories. During training, each sample is kept in the network for 200 milliseconds, during which the teacher layer induces the output layer neuron of the corresponding category to generate spikes. There is an interval of 50 milliseconds between the two samples, during which the coding layer neurons do not generate spikes, which resets the neuron state.

Due to the small number of samples in the IRIS dataset, the hold-out method is implemented to achieve cross-validation during training and validation. Divide each category of data elements into five groups, so that there are 30 data elements in each group, 10 data elements in each category. After dividing the data into five groups, four groups were used for training, and the remaining one group was used for validation. After the training is completed, the remaining group retained in advance is used as the test set to evaluate the network performance.

**FIGURE 6:**
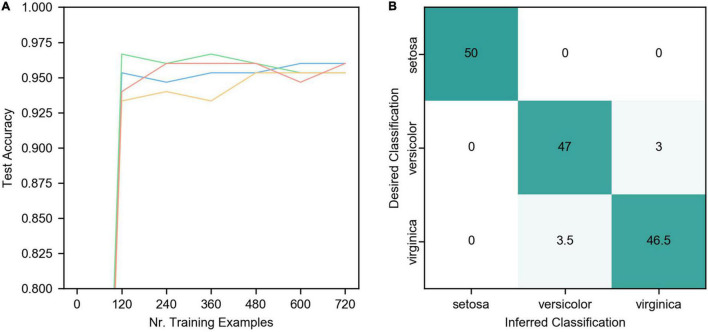
Results of IRIS classification. **(A)** The network accuracy varies with the number of training samples. The four curves represent the data of 4 trials. The other parameters of the four trials are the same except for the random initial weights. **(B)** The final classification confusion matrix, the result is the average of the four trials in **(A)**.

Figure 6 is the result of the IRIS classification network. The detailed network parameters are as follows: 4 groups of coding neurons, 12 in each group, 48 in total, *h* = 20hz, *w* = 2. The synapse weights from the coding layer to the output layer are evenly distributed from 0.2 to 0.3, η = 0.015, α = −0.1, β = 1, τ_p_ = 50, and the teacher spike frequency is 20 hz.

As can be seen from the figure, ALSA can effectively realize the classification of the IRIS dataset. Due to the single-layer structure, there is a certain deviation in the distinction between Versicolor and Virginia. The accuracy of network classification reaches about 95% after learning all training samples (cross-validation, the number of the training set is 120) once, but there are some differences in the four trials. As the number of iterations increases, the accuracy of the four trials gradually converges to a similar value, and the average accuracy of four trials is 95.7%.

### MNIST Classification

The MNIST (LeCun et al., 1998) dataset is widely used in the performance test of various neural networks. The MNIST dataset contains ten classes of handwritten digits from 0 to 9, including a total of 60,000 samples in the training set and 10,000 samples in the test set.

**FIGURE 7:**
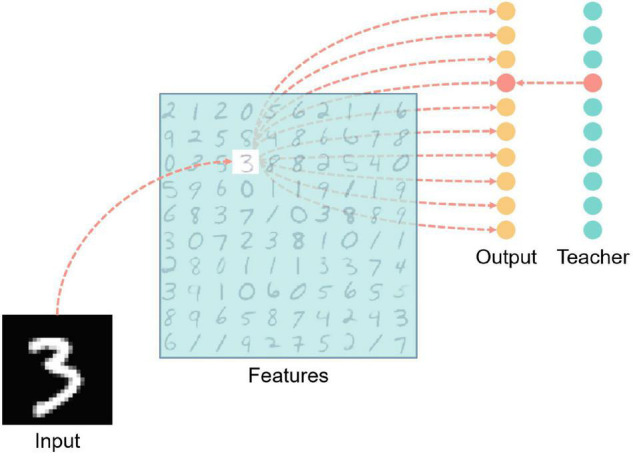
MNIST handwritten digit classification network structure diagram. There are four layers including input, features, output, and teacher. The light blue color blocks in the features layer in the figure indicate inhibition, and the red neurons in the output layer and teacher layer indicate that the neurons are in an active state (that is, there is a spike signal for the time the input vector is presented to the network). The red dashed line indicates the excitatory connection between neurons.

Figure 7 is a structural diagram of the MNIST classification network, including four layers: input layer, features layer, output layer, and teacher layer. The input layer is fully connected with the features layer after the input data is encoded. The encoding method adopts time encoding, that is, the larger the corresponding pixel value, the earlier the neuron spike signal will be emitted, and the spike will not be emitted if the value is lower than the encoding threshold which is a hyper-parameter. Each picture is kept in the network for 200 milliseconds, and there is an interval of 50 milliseconds between two pictures, during which no spike is generated in the input layer, which is for resetting the network state.

Each neuron in the features layer has inhibitory synaptic connections to all neurons in the same layer except itself, which is for achieving lateral inhibition to prevent repeated learning. Also, the features layer neurons are fully connected with the output layer neurons.

Teacher layer neurons are connected one-to-one with corresponding output layer neurons to induce output layer neurons to generate spikes.

The input layer to the features layer is mainly based on unsupervised learning, using the rules of synaptic plasticity described in 6, 7. The training method is similar to Diehl’s work (Diehl and Cook, 2015). For every input image, one neuron in the features layer is activated first and the others are laterally inhibited. The weights between the input layer and the features layer change according to the modified STDP rules. From the features layer to the output layer, ALSA is implemented for supervised learning under the guidance of the teacher layer to realize the mapping of handwritten digitals from the features layer to the output layer.

The network is trained using a layer-by-layer training method, that is, the training between the input layer and the features layer is finished after certain samples, and then the training between the features layer and the output layer is performed.

**FIGURE 8:**
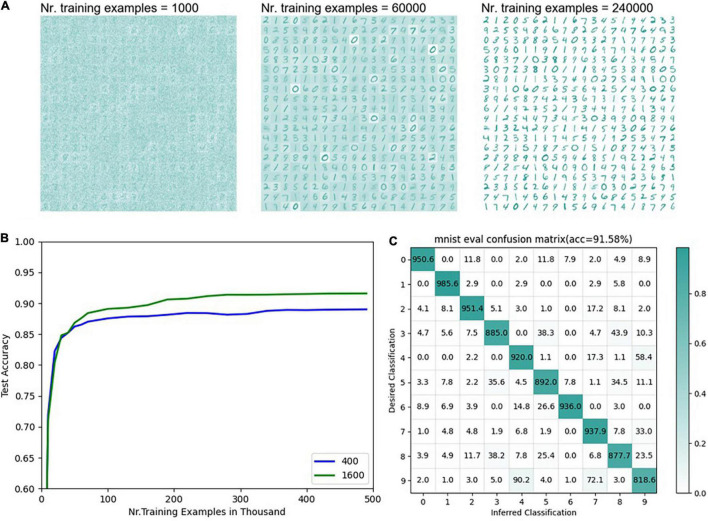
Results of MNIST classification **(A)** The distribution of weights under different numbers of training samples from the input layer to the feature layer with 400 neurons in the feature layer (*n* = 400). **(B)** The accuracy of the supervised part of the network varies with the number of training samples. The blue line represents the accuracy of the model with different numbers of training samples when the number of neurons in the feature layer is 400 (*n* = 400). The green line represents the test accuracy changing with the number of training samples when the number of neurons in the feature layer is 1600 (*n* = 1600). **(C)** The final classification confusion matrix with 1600 neurons in the feature layer.

Figure 8 is the final result of the MNIST classification network. The detailed network parameters are as follows: input layer 28 × 28, consistent with the sample resolution in the MNIST dataset, feature layer 20 × 20, output layer 1 × 10, and teacher layer 1 × 10. The encoding threshold is 0.3. The synapse weights from the input layer to the feature layer are uniformly distributed from 0.01 to 0.11, η = 0.015, α = −0.3, β = 1.3, and τ**_p_** 20. The synapse weights from the characteristic layer to the output layer are uniformly distributed from 0.1 to 0.2, η = 0.001, α = −0.003, β = 2, and τ**_p_** = 100. The teacher spike frequency is 20 hz.

It can be seen from Figure 8A that when there are 400 neurons in the feature layer, as the number of training samples increases, the weights from the input layer to the features layer gradually show the sample pattern clearly. However, there are still some cases where the weight distribution from the input layer to the features layer is fuzzy, or multiple samples are superimposed which is mainly because of lack of learning or small among-class gaps. Samples with smaller intra-class gaps (such as 0, 2, 7) can show clearer contours with fewer training samples. The accuracy of the supervised learning part reaches a higher accuracy after training the complete training samples once, gradually converges in the subsequent training, and finally reaches 88.53%, which has a certain gap compared with the mainstream MNIST classification network. To verify the effectiveness of the supervised learning rules proposed in this paper, it will be compared with the widely cited experimental results of Diehl. It is worth noting that Diehl uses the unsupervised + statistical method in his paper (Diehl and Cook, 2015). In Diehl’s results, when the size of the unsupervised learning output layer is 400 and 1600, the corresponding classification accuracy is 87 and 91.9%, respectively. For the convenience of comparison, this article chooses the network models with 400 and 1600 neurons in the features layer, respectively and the results are obtained in Figures 8B,C. After multiple training runs, the average results are obtained as follows: the classification accuracy of 400 neurons is 88.53%, and that of 1600 excitative neurons is 91.58% (η = 0.065, α = −0.2, β = 1.3, and τ**_p_** = 20). This result indicates that the supervised learning using ALSA in this network can achieve performance similar to the statistical methods of Diehl. It can be seen from the above two classification experiments that ALSA can realize pattern recognition and classification, and it is proved to be working. The feasibility of the ALSA learning method is preliminarily verified here, and more different experiments are needed to improve it in the future.

## Discussion

### Biologically Plausible

The ALSA supervised learning method proposed in this paper is based on associative learning. The synaptic long-term plasticity rule is also based on classic STDP after appropriate modifications. The main contents are supported by corresponding neuroscience-related experiments or phenomena (Krug et al., 1985; Christie et al., 1994; McSweeney and Murphy, 2014). By inducing the target neuron to emit spikes, the connection weights between the neuron corresponding to the current spike pattern and the target neuron are strengthened, and others are weakened, which is consistent with the Hebb rule “neurons that fire together, wire together.” Moreover, the implementation method of supervised learning is similar to the process of animal training based on associative learning, and the latter has been proved to be an effective animal training method in a large number of experiments and practices. Therefore, ALSA is physiologically reasonable and has strong physiological plausibility.

In this paper, a supervised learning algorithm for spiking neural networks based on associative learning named ALSA was proposed. Compared to other supervised learning algorithms for SNN, ALSA is based on modified STDP, thus ALSA is more biologically plausible than most other training algorithms. In addition, the modified STDP used in ALSA shows more similarities to the Hebb rule and actual experiment results in neuroscience. Unsupervised learning is powerful in SNNs due to its great ability in spatial-temporal feature extraction called coincidence detection. Normally, coincidence detection is based on STDP or its modification. While none of the existing supervised learning algorithms excepting ALSA are based on STDP, which make it impossible to realize supervised and unsupervised learning algorithm in the same layer. ALSA shows more compatibility with unsupervised learning algorithms. The key difference of ALSA to unsupervised learning is the teacher signal, without the teacher signal, ALSA works as a normal unsupervised learning algorithm, with the teacher signal, ALSA works as a biologically plausible supervised learning algorithm. Thus, ALSA can make full use of the power of unsupervised learning and supervised learning.

### Compatibility

At present, many SNN supervised learning algorithms have been able to achieve good training effects and performance. But most of the methods are incompatible with unsupervised learning methods. The current unsupervised learning method of SNNs is more reasonable in principle, with stronger physiological plausibility and rationality, and the unsupervised learning method of SNNs has also been proved to have strong feature extraction capabilities, especially the spatial-temporal features extraction ability (Dennis et al., 2015; Masquelier and Kheradpisheh, 2018; Wu et al., 2018). This is an ability that the traditional ANN networks do not have. It is also the place where SNNs have unique advantages. Therefore, it is important to give full play to the unsupervised learning ability of SNNs. ALSA is based on the improved STDP. As shown in the MNIST classification experiment above, this synaptic plasticity rule can realize SNNs unsupervised learning well, that is, the same rule can realize unsupervised learning and supervised learning at the same time. And through appropriate adjustments, in theory, unsupervised learning and supervised learning can be realized in the same layer. Unsupervised learning and supervised learning can be performed at different phases for better learning. Therefore, ALSA has strong compatibility with SNN unsupervised learning, which greatly expands the application scope of ALSA.

### Trainable Layers

Due to the characteristics of ALSA, it can only be used for single-layer network training. However, as mentioned above, ALSA has strong compatibility with SNN unsupervised learning. Therefore, we can make full use of the powerful unsupervised learning ability of SNNs to build multi-layer unsupervised + single-layer supervised SNNs to make up for the shortcomings of only a single-layer training. Also, the supervised method can be used to some key layers in the network by inducing neurons in these layers, to realize multi-layer unsupervised + multi-layer supervised SNNs as a whole.

### Performance

In the experimental part, two experiments, training with IRIS dataset and MNIST dataset are conducted, respectively, and both achieved satisfactory results. The average accuracy of the four training trials of the IRIS dataset was 95.7%. When the number of neurons in the feature layer was 1600, the classification accuracy of the MNIST dataset achieved 91.58%, when training with the proposed ALSA rule. Although the performance achieved by the SNN network has a certain gap compared with the current mainstream ANN networks based on error backpropagation or other classifiers. However, the results of these two experiments prove the feasibility of ALSA to a large extent. In the future, combined with the above-mentioned multi-layer unsupervised and multi-layer supervised methods, with a large network scale, the performance of ALSA has a lot of room for development. Right now, it is still a challenge for us to increase the network scale and improve the recognition accuracy. For the MNIST dataset, there are over 60,000 pictures, and it takes several days to several weeks to train once after further increasing the network scale. In the future, we plan to improve the speed by optimizing the computing framework, such as using multithread or GPU acceleration.

### Robustness

The existence of dynamic membrane potential can prevent some neurons from over-emitting spikes while other neurons do not emit spikes, which will lead to the problem of “winner takes all,” making all neurons have a relatively fair environment to learn spike patterns and improve the efficiency of feature learning. In addition, because of the dynamic threshold, the spiking frequency of teacher neurons has little influence on associative learning and supervised learning. The learning performance of the network is not sensitive to the teacher spiking frequency. According to the results of the two classification experiments, the performance of the final network tends to be stable, indicating that ALSA can control the state of the neural network in a relatively stable state and has high robustness.

## Data Availability Statement

The original contributions presented in the study are included in the article/supplementary material, further inquiries can be directed to the corresponding author.

## Author Contributions

LM proposed the idea and the detailed implementation methods of ALSA. GW designed and implemented the two confirmatory experiments. EL and MZ participated in the revision and supplementary experiment of the manuscript. All authors took part in the writing of the manuscript and discussion of the whole process.

## Conflict of Interest

The authors declare that the research was conducted in the absence of any commercial or financial relationships that could be construed as a potential conflict of interest.

## Publisher’s Note

All claims expressed in this article are solely those of the authors and do not necessarily represent those of their affiliated organizations, or those of the publisher, the editors and the reviewers. Any product that may be evaluated in this article, or claim that may be made by its manufacturer, is not guaranteed or endorsed by the publisher.
